# Dataset on global trade networks of COVID-19 medical products

**DOI:** 10.1016/j.dib.2024.110606

**Published:** 2024-06-10

**Authors:** Marcell T. Kurbucz, Tibor Keresztély, Szabolcs Szikszai, András Sugár, Zsuzsanna Banász

**Affiliations:** a Department of Computational Sciences, Institute for Particle and Nuclear Physics, HUN-REN Wigner Research Centre for Physics, 29-33 Konkoly-Thege Miklós Street, H-1121 Budapest, Hungary; b Department of Statistics, Institute of Data Analytics and Information Systems, Corvinus University of Budapest, 8 Fővám Square, H-1093 Budapest, Hungary; c Department of Economics, University of Pannonia, 10 Egyetem Street, H-8200 Veszprém, Hungary

**Keywords:** Data, Pandemic, International economics, Health goods, Social network analysis, Multilevel network

## Abstract

This paper presents a comprehensive dataset on the global trade dynamics of COVID-19-related medical products for the years 2019 and 2020. The dataset, derived from the BACI database, focuses on eight distinct product categories identified by six-digit codes. The trade flow data for 224 countries is structured as a multilevel network, with countries as nodes and product categories as layers. Directed edges represent trading activities, and edge weights are determined by the difference in exported values between 2019 and 2020. The dataset is provided in an edges-and-nodes format. Additionally, the associated R script transforms the data into the MuxViz R package format, facilitating further analysis and visualization of the dataset. The dataset is valuable for researchers in the field of foreign trade or medical products, and for decision-makers in these fields, whether at company or national level.

Specifications Table

**Table:** 

Subject	Economics, Econometrics and Finance, Data Science, Social Sciences
Specific subject area	Economics, Econometrics, Data Mining and Statistical Analysis, Health
Type of data	Raw data. Comma-separated values files (csv) and an R script (R). Additional files generated after running the R script include edge files (edges) and space- and semicolon-separated files (txt).
Data collection	We obtained international trading data for 2019 and 2020 from the BACI database [1,2]. Utilizing this data, we identified COVID-19-related medical products and their eight categories based on information from the World Integrated Trade Solution's webpage [3]. Subsequently, we calculated the differences in traded values for each product over the investigated two years. Finally, we employed an R script to transform the acquired network into a *MuxViz*-compatible [4,5] multilevel network, with each layer representing one of the eight investigated product categories.
Data source location	Trading data were collected from the BACI database [1,2]. COVID-19-related medical products and their eight categories were identified based on information from the World Integrated Trade Solution's webpage [3].
Data accessibility	Repository name: Mendeley Data Data identification number: 10.17632/b7svp82sdj Direct URL to data: https://data.mendeley.com/datasets/b7svp82sdj Instructions for accessing these data: Freely available on Mendeley Data [6].
Related research article	M.T. Kurbucz, A. Sugár, T. Keresztély, Analysis of the international trade networks of COVID-19 medical products. Appl. Netw. Sci. 8 (2023) 58. https://doi.org/10.1007/s41109-023-00586-z [7]

## 1 Value of the Data

• This dataset provides a comprehensive exploration of global trade dynamics in COVID-19-related medical products for 2019 and 2020, offering valuable insights into the evolution of international connections during this crucial period.
• Focused on eight specific medical product categories identified by six-digit codes, the dataset enables a targeted analysis, providing insights into trade dynamics within distinct sectors.
• With a unique multilevel network structure portraying countries as nodes and product categories (as layers), the dataset visually captures the interconnectedness in trade relationships (as edges), enhancing the understanding of global connections.
• Presented in an edges-and-nodes format (where the nodes are the countries and the edges are the trade between them) with an accompanying R script, the dataset ensures accessibility and facilitates easy transformation into the MuxViz R package, that handles multilevel network format, empowering researchers for in-depth analysis and visualization.
• The dataset is useful for researchers and decision-makers in foreign trade and medical products, at both company and national levels.
• It offers versatile applicability and the MuxViz format, which operates through a user-friendly graphical user interface (GUI).

## 2 Background

Motivated by the unforeseen challenges posed by the COVID-19 pandemic [8], this dataset was created to explore the complexities of global trade networks for medical products during this period. By the end of April 2020, more than 80 countries had introduced export restrictions on critical medical products [9]. The economic and geopolitical impact of COVID-19 and related restrictions on world trade has been examined in a number of studies (see, e.g., [7,10,11]). The aim of this dataset is to deepen our knowledge in this domain by providing a comprehensive dataset in both a simple network and multilevel structure.

## 3 Data Description

### 3.1 Single-layer network format

The dataset consists of two comma-separated values (csv) files, *edges.csv* and *nodes.csv*, which store the edge and node information for a single-layer trading network, respectively. In this network, the nodes represent the 224 countries included in the dataset, and the edges represent the differences in exported values from 2019 to 2020, expressed in thousands of USD. The *nodes.csv* file includes the node ID, the three-letter country codes, and the corresponding geolocations. The *edges.csv* file contains the exporter and importer node IDs, the eight product categories of COVID-19 medical products, as well as the differences in exported values. The variables of both files are detailed in Table 1.

**Table 1:** Single-layer network format.

Variable	Description	Type	Source
*nodeID*	Node (country) ID.	Integer	*nodes.csv*
*nodeLabel*	Three-letter country codes (ISO 3166-1 alpha-3).	String	*nodes.csv*
*nodeLat*	Latitude of the node (country).	Float	*nodes.csv*
*nodeLong*	Longitude of the node (country).	Float	*nodes.csv*
*prodCat*	Product categories. **A**: Medical test kits; **B**: Disinfectants and sterilization products; **C**: Other medical consumables; **D**: Other medical devices and equipment; **E**: Other medical-related goods; **F**: Oxygen therapy equipment and pulse oximeters; **G**: Protective garments; **H**: Vehicles. Further details can be found in the *Data Collection* section.	String	*edges.csv*
*eNodeID*	Exporter node (country) ID.	Integer	*edges.csv*
*iNodeID*	Importer node (country) ID.	Integer	*edges.csv*
*expValDiff*	Differences in exported values from 2019 to 2020 in thousand USD.	Float	*edges.csv*

Table 2 summarizes the exported value differences (*expValDiff*) in each product category (*prodCat*) of the dataset.

**Table 2:** Descriptive statistics of export value differences by product categories.

*prodCat*	Number of Edges	Min	Q1	Median	Mean	Q3	Max	StDev
**A**	11,127	-3.99E+06	-8.71E+00	5.95E-01	2.30E+03	8.11E+01	2.15E+06	5.95E+04
**B**	8,755	-5.65E+06	-9.56E+01	3.61E+00	2.73E+03	5.00E+02	1.73E+06	8.16E+04
**C**	9,989	-2.33E+05	-7.05E+01	-1.05E-01	3.36E+02	5.24E+01	8.27E+05	1.21E+04
**D**	9,875	-6.32E+05	-5.89E+01	1.01E-01	3.24E+02	8.39E+01	1.08E+06	1.88E+04
**E**	8,016	-3.16E+05	-3.31E+01	3.75E-02	2.90E+02	4.21E+01	6.80E+05	1.09E+04
**F**	8,289	-2.13E+05	-2.24E+01	1.40E+00	8.95E+02	1.12E+02	5.29E+05	1.48E+04
**G**	9,390	-5.87E+04	-3.33E+00	3.72E+00	9.48E+03	1.71E+02	1.81E+07	2.36E+05
**H**	4,255	-1.56E+05	-1.85E+02	-2.72E+00	-4.32E+02	4.05E+01	8.24E+04	5.71E+03

According to Table 2, we can classify medical products into three categories based on their exported value growth. The traded value of protective garments (**G**), medical test kits (**A**), and disinfectants and sterilization products (**B**) increased the most. The average increase in these categories was between 2,303 and 9,482 thousand USD. This is followed by categories **C** to **F**, in which the average increase was between approximately 290 and 895 thousand USD. Finally, the international trade of vehicles (**H**) decreased on average during the investigated time period by 432 thousand USD. The most substantial growth observed in the entire dataset occurred within the protective garments (**G**) category, between China and the United States. In this category, China escalated its export value to the United States by a staggering 18,072,670 thousand USD. A detailed analysis of the dataset can be found in Kurbucz et al. (2023) [7].

### 3.2 Multilevel network format

**Fig. 1:**
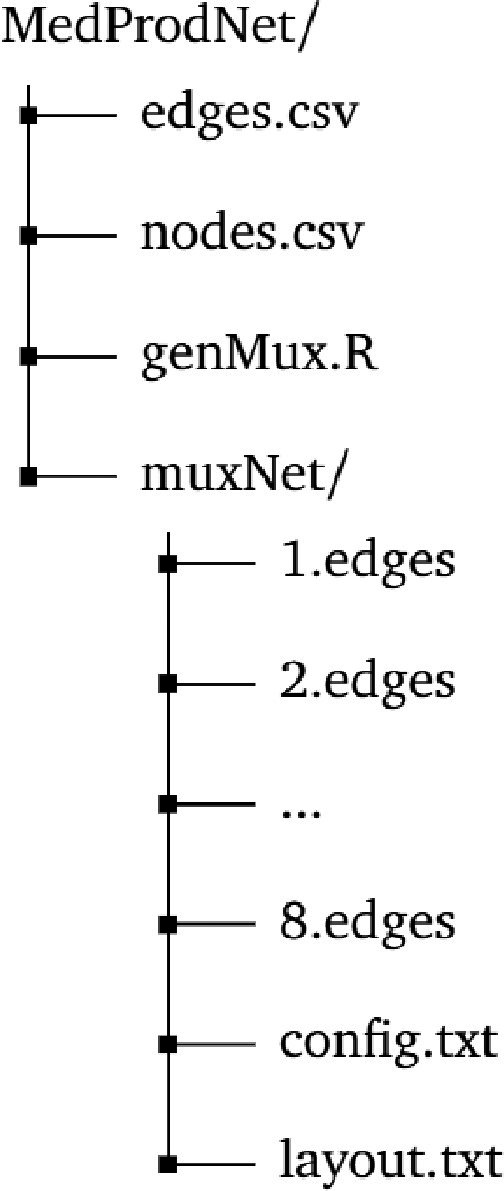
Directory structure.

After running the *genMux.R* script, a new folder named *muxNet* will be generated with the following directory structure (see Fig. 1).

Files contained in the *muxNet* folder do not have headers. Files *1.edges, 2.edges, ..., 8.edges* contain the trade network data for the eight product categories (*A-H*). Each file includes *eNodeID, iNodeID*, and *expValDiff* columns, respectively (see Table 1). The configuration file (*config.txt*) consists of eight rows, each denoting the complete path of an edge file, the title of the edge file (*A-H*), and the complete path of the *layout.txt* file. The layout file (*layout.txt*) includes *nodeID, nodeLabel, nodeLat*, and *nodeLong* columns, respectively (see Table 1).

## 4 Experimental Design, Materials and Methods

### 4.1 Data collection

Data regarding the trade of COVID-19-related medical products is sourced from the BACI database [1,2].^1^ BACI annually provides bilateral trade flow data for 200 countries at the product level. Aligning with Kurbucz [12], our focus is specifically on COVID-19-related medical products identified by six-digit codes (HS-6). These are further categorized into eight distinct product categories as follows [3]:

A: Medical test kits (HS-6: 300215, 382100, 382200, 902780);
B: Disinfectants and sterilization products (HS-6: 220710, 220890, 284700, 300490, 380894, 841920);
C: Other medical consumables (HS-6: 280440, 300510, 300590, 300670, 340111, 340120, 392329, 392690, 481890, 901831, 901832);
D: Other medical devices and equipment (HS-6: 732490, 841319, 901811, 901812, 901890, 902212, 902519, 902780, 902820);
E: Other medical-related goods (HS-6: 731100, 761300, 842139, 940290);
F: Oxygen therapy equipment and pulse oximeters (HS-6: 901819, 901839, 901920, 902680);
G: Protective garments (HS-6: 392620, 401511, 401519, 401590, 481850, 611610, 621010, 621050, 621600, 630790, 650500, 900490, 902000);
H: Vehicles (HS-6: 870590, 871310, 871390).

Aggregate values for exported medical products are grouped by the abovementioned product categories (*A-H*) for the years 2019 and 2020. For each product category, the total exported values from country i to country j in 2019 and 2020 are denoted as $EXP_{i,j}^{2019}$ and $EXP_{i,j}^{2020}$, respectively.

### 4.2 Data processing

The trading data is represented as a multilevel network (see, e.g., [13]). Multilevel networks include multiple layers that can contain a subset of all available nodes and edges. In our case, the eight product categories form eight layers, nodes are the countries, and directed edges represent their trading activities (from the exporting country to the importing country) in the given product category. The weight of the edges within a product category is determined based on the difference between the values exported in 2020 and 2019 as follows:

(1) $$w_{i,j}=EXP_{i,j}^{2020}-EXP_{i,j}^{2019}$$

where $EXP_{i,j}^{2020}$ is the aggregated exported value from country i to j in 2020, measured in thousand United States dollars (USD), i,j∈{1,2,...,L}.

**Fig. 2:**
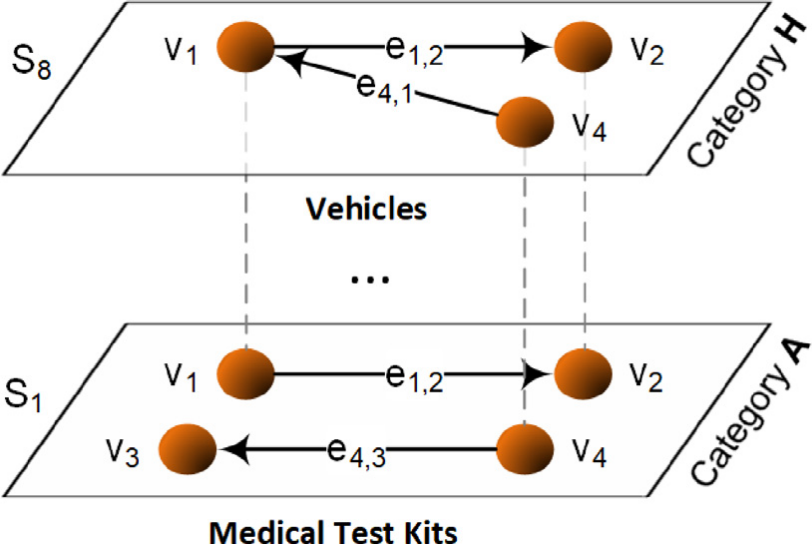
Applied multilevel data structure (Source: Kurbucz et al. [7]).

Formally, the trading data is represented by a graph (G) which is a tuple defined by the sets of nodes (N), edges (E), and eight layers (S) as follows:

G=(N,E,S),

$$S=\left\{S_{1},S_{2},...,S_{K}\right\}\text{sub}-\text{graphs}\;\left(\text{layers}\right),$$

(2) $$\text{with}\;S_{k}=\left(N_{k},E_{k}\right),k\in \left\{1,2,...,K\right\},$$

$$N=\overset{K}{\underset{k=1}{\cup }}N_{k},E=\overset{K}{\underset{k=1}{\cup }}E_{k},$$

where K=8. The applied multilevel data structure is illustrated in Fig. 2.

(Remarks: $v_{i}$: node i, $e_{i,j}$: edge from node i to j. Each $e_{i,j}$ edge has a $w_{i,j}$ weight.)

Note that the directed version of strength (i.e., weighted degree) centrality [14,15] can be employed to assess the magnitude of the change in the total imported and exported values of products for each country between 2019 and 2020, as follows:^2^

(3) $$s_{i}^{in}=\sum_{j=1}^{L}e_{j,i}w_{j,i},\;s_{i}^{out}=\sum_{j=1}^{L}e_{i,j}w_{i,j},$$

where and $\sum_{j=1}^{L}e_{j,i}$ and $\sum_{j=1}^{L}e_{i,j}$ individually measure the in-degree and out-degree centrality of the node i, respectively.

### 4.3 Generating multilevel format

This dataset contains an R script, named *genMux.R*, that transforms the source *edges.csv* and *nodes.csv* into a *MuxViz* [4,5] R package-compatible dataset. During the run, the R script must be in the same folder as the aforementioned two files. This script is operating system-agnostic, and if it is missing, it will automatically install its only dependency, the *rstudioapi* R package.

### 4.4 Illustrative Example

**Fig. 3:**
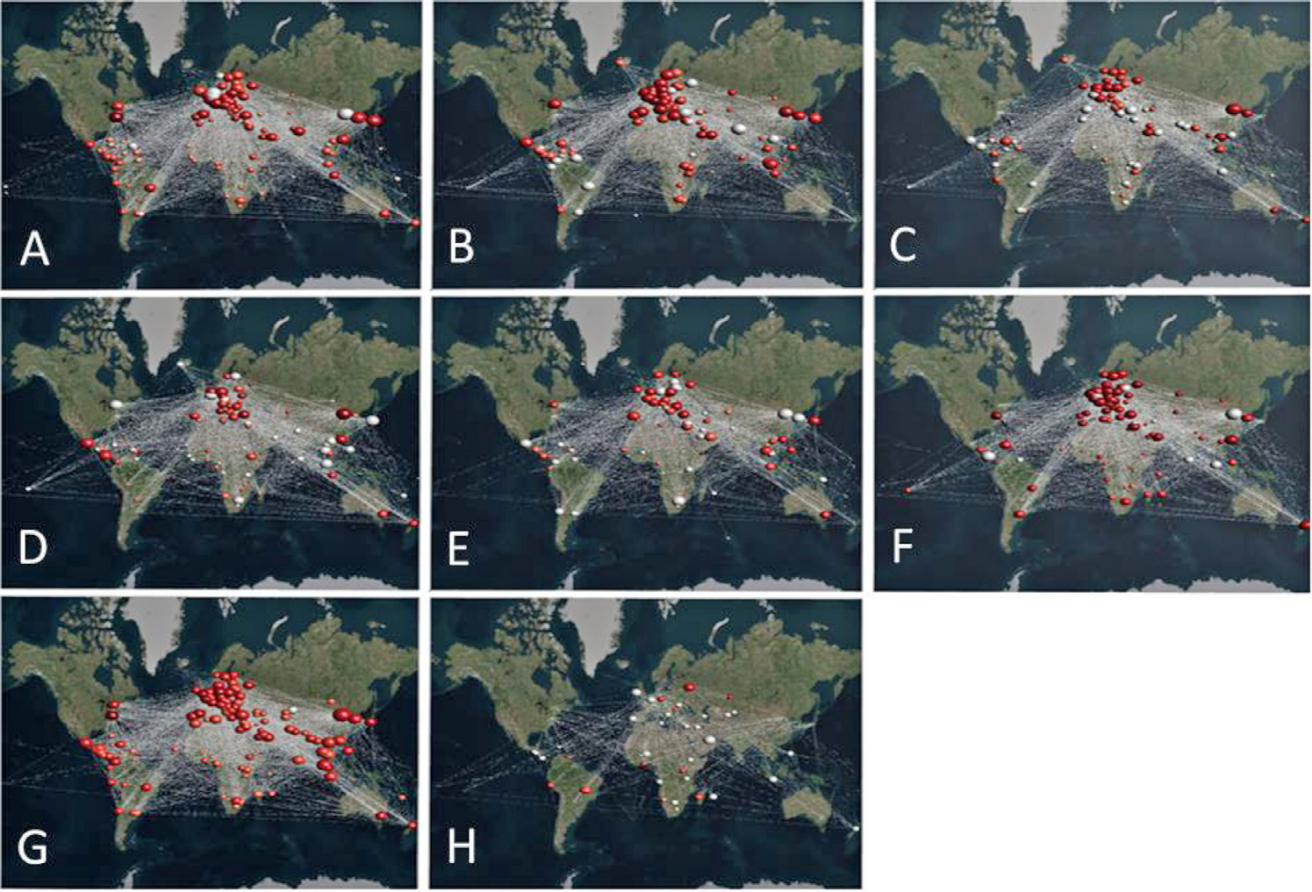
Growth in the import and export of various product categories (A-H), 2019-2020 (Source: Kurbucz et al. [7]).

*MuxViz* [4,5] offers a widespread set of indicators and visualization tools for in-depth analysis of the trading network for COVID-19 medical products. Fig. 3 exemplifies the visualization of strength centralities in *MuxViz*, presented for each layer of the multilayer network separately.^3^

(Remarks: node color: $log(s_{i}^{in})$, from white to red, node size: $log(s_{i}^{out})$, from small to big.)

Fig. 3 uses color coding to represent the change in imports of the eight product categories between 2019 and 2020 ($log(s_{i}^{in})$). White countries indicate a decrease or small increase in imports, while red indicates a relatively large increase. For product categories **A** to **G**, more countries appear red than white, suggesting a general rise in imports. Product category **G** (protective garments) shows the most significant increase, with only a few countries not experiencing a substantial import rise. In contrast, the trade network for healthcare-related vehicles (product category **H**) suggests a global decline in net exports.

Focusing on the size of the country markers, the change in exports of countries in the given product category from 2019 to 2020 is shown ($log(s_{i}^{out})$). Smaller markers indicate a decrease or a relatively smaller increase in exports, while larger markers indicate a larger increase. Across all product categories, Europe and Asia have the largest markers, indicating that these regions exported more products than other continents. Regardless of product category, only a few countries show exceptionally high export values compared to other countries. Among these countries, China showed the greatest increase in net exports for all product categories except **B** and **H**.

## Limitations

Not applicable.

## Ethics Statement

The authors have read and follow the *ethical requirements* for publication in Data in Brief and confirming that the current work does not involve human subjects, animal experiments, or any data collected from social media platforms*.*

## Credit Author Statement

Marcell T. Kurbucz, Tibor Keresztély, Szabolcs Szikszai, András Sugár and Zsuzsanna Banász conceptualized the work and contributed to the writing and editing of the manuscript, as well as data visualization. Marcell T. Kurbucz wrote the software and supervised the research.

## Data Availability

Global Trading Network of COVID-19 Medical Products Between 2019 and 2020 (Original data) (Mendeley Data).

## Footnotes

1 BACI is an abbreviation of the French “Base pour l'Analyse du Commerce International” meaning Database for International Trade Analysis.

2 Strength centrality indicators are available, e.g., in *MuxViz* R package [4,5].

3 Note that Fig. 3 shows the export value differences (*expValDiff*) aggregated to countries.
